# Risk perception of cardiovascular disease among Turkish adults: a cross-sectional study

**DOI:** 10.1017/S1463423623000117

**Published:** 2023-03-27

**Authors:** Sevcan Topçu, Melek Ardahan

**Affiliations:** 1 Nursing Faculty, Ege University, 35030, Izmir, Turkey; 2 Faculty of Health Sciences, Katip Çelebi University, 35620, Izmir, Turkey

**Keywords:** heart diseases, noncommunicable diseases, nursing, perception, risk

## Abstract

**Aim:**

The aim of the study was to determine in adults the risk perception for cardiovascular disease (CVD) and the associated factors.

**Background:**

CVDs are the leading cause of death globally. In adults, perceptions related to the risk for CVDs have a considerable effect on decision-making processes related to one’s own health.

**Methods:**

A cross-sectional study was conducted with 453 adult people from April to June 2019 in İzmir, Turkey. Data were collected with a sociodemographic characteristics questionnaire, perception of risk of heart disease scale (PRHDS), and health perception.

**Findings:**

The mean PRHDS score of adults was 48.88 ± 8.12. The risk perception for CVD was influenced by variables that were age, gender, education, marital status, employment status, health perception, familial cardiovascular disease history, chronic disease status, smoking status, and body mass index. Although CVDs are the most prominent cause of disease-related death in the world, risk perception for CVD was found to be low among the individuals included in this study. This finding indicates the importance of informing individuals about CVD risk factors, raising awareness, and training.

## Introduction

Non-communicable diseases (NCDs) include chronic conditions that are not caused by an infectious process, are non-transmissible, have prolonged courses, are not readily resolved, and do not have a complete cure available. NCDs kill 41 million people each year, equivalent to 71% of all deaths globally (WHO, 2021). The World Health Organization (WHO) explained in “*Ten threats to global health in 2019*” that NCDs, such as diabetes, cancer, and cardiovascular disease (CVD), are among one of the ten global health problems (WHO, 2019). In addition, the burden of disability is primarily driven by NCDs, which were responsible for 80% of disabilities in 2017 (IHME, 2018). Low- and middle-income countries carry the greatest share of these premature deaths, as 85% of NCD-mediated early deaths occur in these countries (WHO, 2021). NCDs are estimated to be responsible for 86% of total deaths in Turkey (Üner, Balcılar, Ergüder, 2018).

NCDs include CVD, cancer, chronic respiratory diseases, and diabetes. CVD is the most important member of these diseases (WHO, 2021). While CVD-associated deaths worldwide were 17.9 million people annually, this number is estimated to increase up to 22.2 million in 2030 (WHO, 2017). Chronic diseases are gradually increasing due to the aging population and changing lifestyles in our country. According to the Turkish Statistical Institute’s (TSI) data, 37.8% of deaths occur due to circulatory system diseases (TSI, 2020). The deaths due to cardiovascular system diseases are most often seen in individuals within 75–84 age group (TSI, 2020). The most important risk factors that cause death and disability in Turkey are tobacco use, high body-mass index, and high blood pressure (IHME, 2018).

Smoking, insufficient physical activity, alcohol consumption, unhealthy nutrition, obesity, hypertension, diabetes, and high blood cholesterol are considered the basic risk factors for CVD (Alissa, 2017; Üner *et al*., 2018). According to the WHO, unhealthy nutrition, insufficient physical activity, smoking, and alcohol consumption represent the most important behavioral risk factors, with consequent effects including high blood pressure, high blood glucose, high lipid levels, extreme weight gain, and obesity (WHO, 2017).

Risk perception is a cognitive process that guides people’s behaviors in the face of situations involving potential risks (Ammouri *et al*., 2011; Pender *et al*., 2015). Risk perception can vary from person to person, and it can affect health-related behaviors, as well as many other aspects of life. In terms of health, risk perception is an important consideration that determines an individual’s commitment to a healthy lifestyle (Dayal and Singh, 2017). According to the Health Belief Model, health behaviors are affected by an individual’s values, beliefs, and attitudes (Pender *et al*., 2015). If individuals believe that a health problem will cause them serious harm, they are aware that the potential for harm will decrease when they take action to reduce the risk. People who perceive themselves to be at risk of negative consequences can regulate risky behavior better than those who do not see themselves as at risk. Risk perception is an important precursor to adopting risk reduction behaviors (Janz and Becker, 1984), and this is affected by different social, cultural, and contextual factors. Therefore, risk perception is a necessary condition for the acquisition of healthy behaviors, but it is not sufficient on its own.

In adults, perceptions related to the risk for CVD have a considerable effect on decision-making processes related to one’s own health. CVD risk perception is one of the most important determinants for individuals to develop and maintain a healthy lifestyle; individuals who do not perceive themselves to be at risk for the development of CVD are unlikely to have behaviors related to a healthy lifestyle (Ammouri *et al*., 2018; Hart, 2005). Individuals with higher CVD risk perception are much more likely to adopt risk-reducing behaviors such as smoking cessation, consistent exercise, and healthy nutrition. A lack of risk perception prevents adults from undertaking protective health behaviors and seeking interventions for the early treatment of CVD (Ammouri *et al*., 2018; Hart, 2005). Therefore, risk perception of CVD is an important feature that should be more rigorously evaluated. The aim of the study was to determine in adults the risk perception for CVD and the associated factors.

## Methods

### Study design and sample

The population of this study was composed of Turkish adults in the province of Izmir, West Turkey (n = 442 839). The sample calculation of this study was performed using the G-Power statistical analysis program (α:0.05, β:0.05, and d:0.5). According to Cohen (1988), a medium effect size was preferred in the sample calculation. The sample size required for this study was determined to be 423. After including a nonrespondent rate of 10%, the sample size was calculated as 465.

The criteria for participation were as follows: (a) adult age (18 years or older), (b) ability to communicate in Turkish languages, and (c) ability to provide informed consent. A questionnaire was asked to 465 participants. There were 12 participants who did not answer some of the questions and were, therefore, excluded from the survey. The remaining 453 (%97.4) participants completed the questionnaire in full and were included in this study.

### Data collection

Data were collected in the Bornova district of İzmir province an area that ranks second in deaths caused by CVD according to Turkish statistics through face-to-face survey method from April to June 2019. This district is an old residential area consisting of 45 neighborhoods where 442 839 people live. The nine neighborhoods where 5102 adults live in (18 years old and over) were randomly selected for this study considering the rate of not responding to the face-to-face surveys and the possibility that potential participants could not be reached for several reasons (not being at home, employment, etc.). Face-to-face surveys were held in households in nine neighborhoods. Face-to-face surveys were preferred to increase the response rate.

Data were collected by two researchers after the study’s purpose was explained to the potential participants. Surveys were started with the first adult resident contacted at a random sample of residences for every selected neighborhood and were continued with the nearest household to this. Each survey was completed in 10–15 min. Researchers returned to the same neighborhoods an average of six times until reaching the required sample size.

### Instruments

The data collection tools the sociodemographic characteristics questionnaire, the perception of risk of heart disease scale (PRHDS), and health perception were used.

The sociodemographic characteristics questionnaire consisted of total 11 questions: age, gender, education status, marital status, employment status, income level, chronic disease status, familial CVD history, smoking status, height, and weight. As recommended by the WHO, participants were classified according to body mass index (BMI) as follows: <18.5 underweight, 18.5–24.9 normal weight, 25.0–29.9 overweight, 30.0–34.0 obesity class I, 35.0–39.0 obesity class II, and above 40.0 obesity class III (WHO, 2010).

Ammouri and Neuberger (2008) developed the PRHDS to determine the risk of individual CVD. PRHDS is a 4-point Likert scale consisting of 20 items and three subscales. Dread risk is defined at its high end as perceived dread, catastrophic potential, lack of control, and fatal consequences. Risk is reflected as a hazard that has a few moderate, known outcomes and consequences. Unknown risk is defined at its low end as the perception of hazards judged to be unobservable, unknown, new, and delayed in their manifestation of harm (Ammouri and Neuberger, 2008). The scores that can be taken from the scale vary between 20 and 80. Risk perception level increases as the scores obtained from scale are increased. The scale’s Turkish validity-reliability study was carried out by Toptaner (2013). Cronbach’s alpha coefficient was 0.80 (Toptaner, 2013). In this study, Cronbach’s alpha coefficient was 0.84.

Health perception was rated by adults and measured with five point Likert Scale (very good, good, fair, poor, and very poor).

### Data analyses

Data were analyzed using IBM Statistical Package for Social Sciences (SPSS version 20.0). Sociodemographic characteristics reported as frequencies, mean, and percentages. Multivariate regression analysis was utilized for evaluation of PRHDS, dread risk, risk and unknown risk, and affective factors

### Ethical considerations

In order to carry out the study, permission was obtained from University Medical Research Ethics Committee (Approval Number:19-4T/45). Written informed consent was obtained from the persons who agreed to participate in the study.

## Results

Participants’ age ranged from 18 to 86 (M = 38.53 ± 13.42). Of the sample, 53.9% were women, 66.4% were married, 76.2% were employed, and 56.1% had bachelor’s degree (Table 1).

**Table 1.** Sociodemographic characteristics

Variables	Participants (N = 453)	
	n	%
**Age (years; M and SD)**	38.53	13.42
**Gender**		
Man	209	46.1
Woman	**244**	**53.9**
**Marital Status (%)**		
Married	**301**	**66.4**
Single	152	33.6
**Education Status (%)**		
Literate	12	2.6
Elementary School	76	16.8
Junior High School	111	24.5
University and above	**254**	56.1
**Vocational Status (%)**		
Working	**345**	**76.2**
Non-working	108	23.8
**Income Status (%)**		
Income equal to expense	**298**	**65.8**
Income more than expense	75	16.6
Income less than expense	80	17.7
**Smoking Status (%)**		
Smoke	126	27.8
Don’t Smoke	**282**	**62.3**
Quit smoke	45	9.9
**Chronic Disease Status**		
Yes	90	19.9
No	**363**	**80.1**
**Familial CVD History**		
Yes	186	41.1
No	**267**	**58.9**
**Self-Rated Health Perception**		
Very good	**59**	**13**
Good	**248**	**54.7**
Fair	**137**	**30.2**
Poor	**8**	**1.8**
Very poor	**1**	**0.3**
**Body Mass Index**		
<18.5	14	3.1
18.5–24.9	209	46.1
25.0–29.9	185	40.8
≥30	45	9.9

The mean PRHDS score of adults was 48.88 ± 8.12, the mean of dread risk subscale score was 16.09 ± 4.34, the mean of risk subscale score was 15.22 ± 3.22, and the mean of unknown risk subscale score was 17.55 ± 3.49 (Table 2).

**Table 2.** PRHDS and Subscale score of participants (N = 453)

	X ± SS (min–max)	Possible Range
PRHDS	48.88 ± 8.12 (22–74)	20–80
Dread Risk	16.09 ± 4.34 (7–28)	7–28
Risk	15.22 ± 3.22 (6–23)	6–24
Unknown Risk	17.55 ± 3.49 (7–28)	7–28

PRHDS and subscales, age, gender, education, marital status, employment status, health perception, familial CVD history, chronic disease status, smoking status, and BMI were assessed using regression analysis. At the end of regression analysis, a statistically significant was detected between these variables and risk perception for CVD (R^2^ = 0.27, *P* < 0.01), dread risk (R^2^ = 0.22, *P* < 0.01), risk (R^2^ = 0.17, *P* < 0.01), and unknown risk (R^2^ = 0.19, *P* < 0.01). This model showed that the variables were powerful predictors of perception of CVD. The risk perception of CVD was affected by age, education, marital status, employment, health perception, presence of familial CVD history, chronic diseases status, smoking status, and BMI (*P* < 0.05). Age, marital status, employment, health perception, presence of familial CVD history, and chronic disease status had a significant association with the dread risk subscale (*P* < 0.05). In addition, also the variable of age, gender, education, marital status, employment, health perception, and smoking had affected risk subscale statistically significantly; age, education status, employment, health perception, smoking, and BMI variables had affected unknown risk subscale statistically significantly (*P* < 0.05) (Table 3).

**Table 3.** Multiple regression analysis of demographic variables on perception of risk of CVD (N = 453)

Variables	PRHDS		Dread Risk		Risk		Unknown Risk	
	β	p	β	p	β	p	β	p
Age	0.24	0.01	0.14	0.02	0.25	0.01	0.16	0.01
Gender	0.12	0.01	0.08	0.11	0.13	0.01	0.07	0.20
Education	0.12	0.03	−0.02	0.59	0.11	0.04	0.21	0.01
Marital Status	0.15	0.01	0.10	0.04	0.16	0.01	0.09	0.06
Employment Status	0.27	0.01	0.14	0.02	0.24	0.01	0.22	0.01
Health Perception	−0.21	0.01	−0.22	0.01	−0.13	0.01	−0.11	0.02
Familial CVD History	0.14	0.01	0.18	0.01	0.07	0.15	0.04	0.43
Chronic Disease Status	0.12	0.01	0.14	0.01	0.03	0.56	0.08	0.12
Smoking Status	−0.10	0.02	0.04	0.35	−0.12	0.01	−0.17	0.01
BMI	−0.11	0.02	0.00	0.95	0.06	0.23	−0.20	0.01
R	0.52		0.47		0.42		0.44	
R^2^	0.27		0.22		0.17		0.19	
F	16.11		12.53		9.21		10.6	
p	0.01		0.01		0.01		0.01	

*p<0.05.

## Discussion

In this study, the risk perception for CVD and the associated factors was to determine in adults. Previous studies have documented that the risk perception for CVD is not at the desired level. For example, Dayal and Singh (2017) surveyed a group of 20- to 40-year-old adults and found that more than half of the participants assumed themselves at risk of heart disease in the future, with a medium score of risk perception for CVD. In another study conducted on 300 Jordanians adults, Ammouri *et al*., (2011) found that despite the fact that CVD is a major cause of death in Jordan, participants had a medium score of risk perception for CVD. Also, the same study stated that there was a need for heart disease education programs for all adults. Johnson *et al*. (2015) carried out a study among 174 adults ages 40–79 years old who had three or more basic CVD risk factors and found that participants had a high mean score of risk perception for CVD (56 out of 80). Although CVD is the leading cause of death in Turkey, this study found Turkish individuals to have a insufficient level of perceived risk for this disease, which is consistent with similar studies (Ammouri *et al*., 2011; Dayal and Singh, 2017). An understanding of the participants’ perception of CVD risk is important in reducing the associated morbidity and disability rates. The results of this study show that adults ignore the risk of CVD. The basis of the fight against CVD is the detection and prevention of risk factors, including smoking, being overweight or obese, insufficient physical activity, and an unhealthy diet. Risk perception is an important precursor to engaging in preventive behaviors and making appropriate changes (Janz and Becker, 1984). Several other studies found that the level of perceived risk for CVD increased when individuals were provided with education about risk factors or when these were discussed during individual consultations (Toptaner, 2013; Bustanji and Majali, 2013). Therefore, particular emphasis should be placed on informing individuals about CVD risk factors.

Aging is a nonmodifiable risk factor for CVD. That means as age increases, the risk of CVD also increases (Zipes *et al*., 2018). Dayal and Singh (2017) found that age had a strong and significant association with dread risk and risk subscales. In this study, increased age was associated with higher PRHDS, dread risk, and unknown risk. These results showed that increased age made individuals more aware of the risk of CVD.

Malyutina *et al*. (2004) reported that the mortality rates of cardiovascular and coronary heart diseases were higher in single women and men compared to their married counterparts. Many studies have established that CVD risk and mortality rates are lower in married people compared to single individuals and have also found that single men were the population at greatest risk for CVD and associated death (Eaker *et al*., 2007; Manfredini *et al*., 2017). In this study, the mean PRHDS, dread, and risk subscale scores were lower in single compared to married individuals. Although single individuals are known to be at greater risk for CVD, this population does not tend to consider themselves at risk.

In a study by Tillmann *et al*. (2017), low education level was identified as a causal risk factor for the development of coronary heart disease. Ammouri *et al*. (2011) reported that the risk perception for CVD was higher in individuals with higher education levels. Similarly, in this study found that education level affected risk perception for CVD, and risk, unknown risk subscales, and PRHDS were correlated with education level. These data suggest that individuals with higher education levels are better able to understand and evaluate CVD risk factors.

Employment status is another influential factor for risk perception of CVD. Dayal and Singh (2017) reported that employment status was associated with PRHDS score, and risk perception for CVD was higher in retired individuals compared to other groups. In this study, PRHDS, dread risk, unknown risk, and risk were found to be higher in working individuals than other populations.

Health perception is a basic concept that determines the realization and maintenance of preventive health behaviors (Pender *et al*., 2015; Saleh *et al*., 2019). Several studies showed that individuals who had worse health perception had higher mortality risks related to CVD (Stenholm *et al*., 2016; Waller *et al*., 2015). However, health perception is not always compatible with people’s current health status (Pender *et al*., 2015). Holt *et al*. (2020) found that perceived CVD risk was significantly higher among participants who rated their general health as fair or poor. Similarly, in this study, worse health perception was significantly associated with higher PRHDS and subscale scores. These results suggest that health perception is a very effective predictor for perceived risk of CVD. Therefore, nurses should absolutely evaluate health perception when discussing CVD risk factors with adults.

Petitte (2018) found that university students who interview their grandmother/grandfather about their CVD showed increased risk perception scores for CVD. In addition, individuals with a familial CVD history and those having numerous family members with current disease showed a higher risk perception for CVD and increased perception of dread risk compared to other groups. In the present study of adults, the presence of chronic diseases, and knowledge of familial CVD history increased the risk perception of CVD and dread risk.

Obesity (Kalyoncuoğlu *et al*., 2017) and smoking (Lemos and Omland, 2017) are two fundamental CVD risk factors. Smoking doubles the CVD risk (Lemos and Omland, 2017). Smoking cessation is considered to be the most effective protective measure for CVD (Lemos and Omland, 2017). A study seeking to evaluate current factors related to NCDs in Turkey found that 47.8% of participants had 1 or 2 risk factors related to CVD (Üner *et al.,* 2018). In this study, increased BMI was associated with lower PRHDS and unknown risk for CVD, whereas smoking decreased the PRHDS and perception of risk and unknown risk. Individuals who do not consider themselves at risk for CVD do not attempt to prevent or control the disease, even though they may possess some risk factors (Ammouri and Neuberger, 2008).

The results of this study demonstrate that many individuals do not believe themselves to be at risk of CVD despite having important risk factors such as smoking and being overweight or obese. Even smokers had a lower level of perceived risk of CVD while also being afraid of it. Recent studies show that underestimating risk factors (Soroush *et al*., 2017), poor risk perception (Saeidi and Komasi, 2018), and unhealthy lifestyles (Chu *et al*., 2016) are among the main causes of increased CVD risk. Risk perception plays a prominent role in the prevention of CVD by increasing readiness for lifestyle changes (Barnhart *et al*., 2009). Ammouri *et al*. (2011) state that when people compare themselves to others with similar characteristics, such as age, sex, eating habits, working conditions, and lifestyle habits, they tend to underestimate their own risk factors. Therefore, participants do not perceive themselves to be at risk despite having certain risk factors.

## Limitations

This study has some limitations. Self-reporting was used to evaluate risk perception of CVD and weight–height-related measurements. Therefore, a social desirability bias could exist. The study was a cross-sectional study, and in future studies, should be employed a longitudinal design to confirm the findings, and investigate the causality of relationships.

## Conclusion

Although CVD is the most prominent cause of disease-related death in world, risk perception for CVD was found to be low among the individuals included in this study. The risk perception for CVD disease was influenced by diverse variables including age, gender, education, marital status, employment status, health perception, familial CVD disease history, chronic diseases, smoking, and BMI.

Even if healthy individuals do not yet have a disease, they may be at risk of certain diseases due to their family history, eating habits, and lifestyle behaviors. An understanding of society’s perception of CVD risk and the variables that affect this is imperative in raising awareness of potential risks and preventing disease. Thus, individuals with a high risk of CVD but a low level of perceived risk can be identified, and the necessary interventions can be implemented.

When the economic burden of CVDs is evaluated, the recommended fundamental strategy is primary protection, which involves disease prevention and risk evaluations. More training should be given by nurses to increase community awareness of CVD and its risk factors and to reduce CVD morbidity and mortality rates. Community-based screening programs that include risk factors such as blood pressure and lipid levels should be provided and training opportunities that encourage individuals to undertake protection measures should be implemented by public health nurses who are in direct contact with the community.
